# *EGFR*突变与非小细胞肺癌放射治疗进展

**DOI:** 10.3779/j.issn.1009-3419.2013.03.08

**Published:** 2013-03-20

**Authors:** 幸 钟, 瑾 王

**Affiliations:** 610041 成都，四川大学华西医院肿瘤中心胸部肿瘤科 Division of Thoracic Tumor, Cancer Centre, West China Hospital, Sichuan University, Chengdu 610041, China

**Keywords:** 放射治疗, 肺肿瘤, 表皮生长因子受体, 突变, Radiotherapy, Lung neoplasms, Epidermal growth factor receptor, Mutation

## Abstract

放射治疗在肺癌治疗中占据重要地位。表皮生长因子受体（epidermal growth factor receptor, EGFR）突变是晚期非小细胞肺癌（non-small cell lung cancer, NSCLC）使用酪氨酸激酶抑制剂（tyrosine kinase inhibitor, TKI）治疗有效的预测因子。同时，*EGFR*突变型NSCLC对放射治疗敏感，这可能与突变型*EGFR*不能有效进行核转位而导致DNA损伤修复功能受损相关。初步研究显示EGFR酪氨酸激酶抑制剂在NSCLC放射治疗中具有一定的放疗增敏作用，但其在*EGFR*突变型NSCLC放疗中的疗效尚不确切。*EGFR*突变与NSCLC的放疗效应机制及生存预后的关系值得进一步研究。

## Abstract (en)

Radiotherapy plays a pivotal role in the treatment for lung cancer. Epidermal growth factor receptor (*EGFR*) mutation in non-small cell lung cancer (NSCLC) which predicts tyrosine kinase inhibitor (TKI) treatment response may also has effect on radiation response. NSCLC harboring kinase-domain mutations in EGFR exhibits enhanced radio-sensitivity due to dramatically diminished capacity to resolve radiation-induced DSBs (DNA double-strand breaks) associating with the inefficiency of EGFR nuclear translocation. Recently, several preliminary clinical studies show certain efficacy of concurrent EGFR tyrosine kinase inhibitors and radiotherapy. However its further response in *EGFR*-mutated NSCLC is unclear. The correlation between *EGFR* mutation genotype and the radiotherapy response and clinical outcome is worthy of further study.

肺癌为人类恶性肿瘤的发病率、死亡率最高之一，尽管治疗手段不断提高，但目前非小细胞肺癌（non-small cell lung cancer, NSCLC）患者5年生存率仍不足15%^[1]^。

近年来肺癌分子生物学研究取得突破性进展，针对小分子靶点的抗肿瘤药物为NSCLC患者带来福音。其中以表皮生长因子受体（epidermal growth factor receptor, EGFR）为靶点的酪氨酸激酶抑制剂（tyrosine kinase inhibitors, TKIs）最为突出。然而TKI药物在NSCLC患者中有效率相差甚大，其中，在EGFR基因突变阳性的患者中TKI治疗有效率高达70%-80%，明显延长患者无进展生存时间及生活质量，其中位生存时间达20个月-30个月^[2-5]^。因此，EGFR酪氨酸激酶编码区基因突变成为TKI药物奏效的重要靶标^[6]^。

放射治疗在NSCLC的治疗中占有重要地位，而*EGFR*突变对肺癌放疗的效应机制尚未完全明了, 因此针对不同表型EGFR对放疗敏感性影响的个体化治疗是目前肺癌研究的方向。本文就NSCLC中*EGFR*突变状态与放疗敏感性的关系作一综述。

## 1 *EGFR*突变与NSCLC

### 1.1 EGFR家族

*EGFR*基因定位于人第7号染色体短臂，由118 kb组成，包括28个外显子，其中第18-21号外显子编码酪氨酸激酶区域。其表达产物EGFR是由单一多肽链组成的跨膜糖蛋白，激活后可启动Ras/Raf/MEK/ERK、PI3K/AKT、STAT等途径，参与促细胞生长分化、血管形成、调控DNA损伤的修复、抗凋亡、诱导细胞周期阻滞等生物过程^[7]^。

### 1.2 EGFR基因突变

在NSCLC中，*EGFR*突变总发生率约为26%^[8]^。突变多发生在外显子18-21区，即酪氨酸激酶区，这可能与该区域DNA富集A-T序列有关^[9]^。EGFR存在突变蛋白：EGFR Ⅵ、EGFR Ⅱ和EGFR Ⅲ，EGFR Ⅷ是最常见的突变型，其突变形式为胞外氨基酸残基缺失，导致EGFR失去了配体结合域且受体酪氨酸激酶处于持续活化状态。因此，*EGFR*突变常被视为原癌基因的“激活突变”。

其中90%的突变位于19和21区的外显子，称为经典型突变，外显子18和20突变约占10%^[10]^。外显子19的突变（约占45%）主要是第746-750位密码子（ΔE746-E750）缺失突变，导致其相应的氨基酸序列丢失；此外，近年新报道外显子19还存在少见的插入突变，为第2212-2234位核苷酸插入性突变，导致6个相应氨基酸序列插入^[9]^。外显子21的点突变（约占45%-50%）则为第858位密码子（L858R）出现T-G转换，而使得该位点的亮氨酸变为精氨酸。这3种突变均能使其受体的酪氨酸激酶ATP结合槽（ATP-binding cleft）发生变构，导致其结合ATP能力下降，从而增强肿瘤细胞对TKIs的亲和力和反应性，成为TKI药物奏效的重要靶标^[10]^。此外，*EGFR*突变高度激活Ras/Raf/MEK/ERK和P13K/AKT下游通路，启动EGFR调节抗凋亡和生存信号，导致肿瘤细胞变得依赖此信号以维持其生存——即具有癌基因（突变的*EGFR*）依赖的特征（oncogene addiction模型）；当使用特异性TKI阻断EGFR信号后，便能更大程度地抑制其增殖性影响和输出生存信号，导致肿瘤细胞死亡^[11, 12]^。但是，目前鲜有数据显示外显子18、20突变带来TKI获益，反而，外显子20T790M位点突变成为TKI耐药的重要机制之一^[8]^。

## 2 EGFR突变与NSCLC放射敏感性

### 2.1 *EGFR*突变型NSCLC对放疗敏感

在NSCLC中，大约80%鳞癌及65%腺癌存在EGFR蛋白过表达，而这种高表达状态是导致NSCLC放疗抵抗的重要因素^[13-15]^。*EGFR*突变型NSCLC常常伴随*EGFR*基因拷贝数的扩增及蛋白的高表达^[16, 17]^。

然而，Das等^[18]^在体外试验发现，*EGFR*野生型NSCLC细胞对放射抗拒，*EGFR*突变型细胞却对放射敏感。研究显示，相比野生型，*EGFR*突变型细胞受照射后的存活率明显下降，伴随凋亡及核内DNA碎片明显增加。将突变型*EGFR*基因片段（ΔE746-E750缺失或L858R替换突变）转染人支气管上皮细胞（humar bronchial epithelial cell, HBEC）、A549及H1299细胞株后均可提高其放射敏感性，而转染野生型*EGFR*基因片段后其放射敏感性明显降低，进一步提示了*EGFR*突变与放射敏感性相关。

另外，针对NSCLC患者的放疗疗效分析也证实了这一观点（表 1）。2008年Gow等^[19]^回顾性分析了63例NSCLC脑转移患者*EGFR*突变状态与全脑放疗（whole brain radiation therapy, WBRT）疗效的关系。此研究显示，*EGFR*突变型患者WBRT有效率（54%）明显高于野生型患者（24%），并提出EGFR突变和TKI治疗是此类患者WBRT疗效的独立预后因素。2011年，哈佛医学院放疗中心对123例既往行单纯放疗或放疗联合其它治疗（化疗、手术、靶向治疗）的局部晚期NSCLC患者进行回顾性疗效分析发现，*EGFR*突变型患者放射治疗后2年内局部复发率（17.8%）要明显低于*EGFR*野生型患者（41.7%），同时*EGFR*突变型患者2年生存率（92.6%）也要明显高于野生型患者（69.0%）。多因素分析^[20]^显示*EGFR*突变是降低放疗后局部复发率（locoregional recurrence rate, LRR）的独立影响因素。此外，Lee等^[21]^报道，在43例NSCLC脑转移患者全脑放疗后，*EGFR*突变型患者的局部无疾病进展生存（radiological progression-free survival, RPFS）要明显高于EGFR野生型患者（21个月*vs* 12个月）。

**1:** *EGFR*突变与NSCLC放疗疗效的回顾性临床研究结果 Retrospective analysis on *EGFR* mutation and radiation treatment outcome for NSCLCs

Reference	Study type	Patients	RT schedule	Systemic therapy	Result			Md OS (month)	
					WT	Mutated		WT	Mutated
Gow CH. *et al* ^[19]^	PhaseⅡ single institution (*n*=63)	Lung adenocarcinoma with brain metastases	WBRT 30 Gy-35 Gy/ 15 f-18 f	With or without TKI	RR (%): 24 (4/17) 54 (25/46) *P*=0.045			6.6 17.3 *P*=0.121	
Mak RH. *et al* ^[20]^	PhaseⅡ single institution (*n*=123)	NSCLC stage Ⅱb-Ⅲb No prior thoracic RT	Curative RT 41.4 Gy-74 Gy	With or without chemotherapy /surgery	LRR (%): 41.7 (39/94) 17.8 (5/29) *P*=0.005 2-year RFS%: 35.8 41.4 *P*=0.33			34.7 61.2 *P*=0.05	
Lee HL. *et* *al* ^[21]^	PhaseⅡ single institution (*n*=43)	NSCLC with brain metastases	WBRT 30 Gy-40 Gy/15 f-20 f, 17 cases local boost to 50 Gy-60 Gy	With or without TKI	RR (%): 46 (6/13) 80 (24/30) *P*=0.037 RFS (month): 21.0 12.0 *P*=0.009			15.0 11.0 *P*=0.049	
EGFR: epidermal growth factor receptor; NSCLC: non-small cell lung cancer; TKI: tyrosine kinase inhibitor; RT: radiation therapy; WBRT: whole brain radiation therapy; RR: response rate; LRR: locoregional recurrence rate; RFS: relapse-free survival; WT: EGFR wild type; Mutated: *EGFR* mutated type; Md OS: median overall survival.									

虽然目前针对*EGFR*突变对放疗疗效影响的研究多为Ⅱ期回顾性试验，混杂因素较多，但仍提示我们*EGFR*突变可能成为预测晚期NSCLC患者放疗疗效的重要指标。

### 2.2 *EGFR*突变影响NSCLC放射敏感性机制

EGFR通过参与NSCLC细胞对放射致死性损伤（DNA double-strand break, DSB）修复而导致放疗抵抗。激活的EGFR主要通过参与非同源末端连接（non homologous end-joining, NHEJ）和同源重组修复（homologous recombination, HR）途径对DNA损伤进行修复^[8]^。

Das等^[22]^进一步研究发现*EGFR*突变型NSCLC的放射敏感性与其NHEJ功能缺陷密切相关。电离辐射后，*EGFR*突变细胞株及异位表达突变*EGFR*基因的HBEC细胞均不能有效进行EGFR核移位，致使EGFR无法与DNA-PK修复蛋白（DNA-PKcs、Ku等）结合，进而大大削弱了其NHEJ途径对DNA损伤的修复能力而增加其放射敏感性。突变型*EGFR*依赖的Ras/Raf/MEK/ERK和PI3K/AKT通路是同源重组修复（homology recombination, HR）的重要途径，但目前尚无针对*EGFR*突变与HR关系的相关研究。此外，在*EGFR*野生型与突变型NSCLC细胞中，DNA损伤修复蛋白（如DNA-PKcs、Ku70/80、Rad51等）在表达水平或功能上是否存在差异也不清楚。这些值得我们进一步研究。

## 3 TKI联合放疗的研究进展

体外试验^[23-26]^显示，使用酪氨酸激酶抑制剂TKI（厄洛替尼、吉非替尼）可通过增加细胞周期阻滞及凋亡、下调Rad51蛋白等途径增加的NSCLC的放射敏感性。台湾的一项研究^[27]^显示，在一线使用TKI治疗有效的Ⅲ期/Ⅳ期NSCLC患者中，及早联合放疗治疗可获得较好的疗效，其中总体有效率为84%，中位无进展生存期（progression-free survival, PFS）为16个月，3年生存率达62.5%。另一项研究^[28]^回顾性分析了吉非替尼联合全脑放疗对比吉非替尼单药治疗NSCLC脑转移患者的疗效，联合治疗组在总生存期（overall survival, OS）上明显获益（23.4个月*vs* 14.8个月）。此外，一项前瞻性研究显示，在25例Ⅲ期/Ⅳ期NSCLC患者中，放疗联合使用TKI（吉非替尼或厄洛替尼）治疗的局部控制率达96%，中位PFS、OS分别为10.2个月和21.8个月^[29]^（表 2）。由于对TKI有效的NSCLC人群多存在*EGFR*突变，因此这些研究显示的TKI联合放疗的优效性可能与*EGFR*突变相关。

**2:** TKI联合放疗治疗NSCLC的临床研究结果 Clinical trials on TKI combined with radiation treatment for NSCLCs

Reference	Study type	Patients	RT schedule	Systemic therapy	Result	Survival (month）
Chang CC. *et al* ^[27]^	Retrospective Single institution (*n*=25)	NSqCLC stage Ⅲb or Ⅳ responded to TKI	Hypofractionated RT 40 Gy-50 Gy /16 f-20 f	With TKI	RR (%): 84.0	Md PFS: 16.0
Zeng YD. *et al* ^[28]^	Retrospective Single institution (*n*=90)	NSCLC with brain metastases	With or without concomitant WBRT	With TKI	RR (%): RT+TKI 64.4 TKI 26.7 *P* < 0.001	Md OS: RT+TKI 23.4 TKI 14.8
Wang J. *et* *al* ^[29]^	Prospective Single institution (*n*=25)	NSCLC stage Ⅲb or Ⅳ	Curative RT: 66 Gy/33 f	With TKI	LCR (%): 96.0	Md PFS 10.2 Md OS 21.8
Ready N. *et al* ^[31]^	Prospective Single institution (*n*=60)	NSCLC stage Ⅲ	Curative RT: 66 Gy/33 f Poor-risk: concomitant RT Good-risk: concurrent CRT	2 cycle introduction chemotherapy followed by TKI	RR (%): RT+TKI 52.4 CRT+TKI 81.6	^*^Md PFS: RT+TKI 13.4 CRT+TKI 9.2 ^*^Md OS: RT+TKI 19.0 CRT+TKI 13.0
Md PFS: median progress free survival; NSqCLC: non-squamous cell, non-small cell lung cancer; LCR: local control rate; ^*^: no apparent survival difference with EGFR-activating mutations versus wild type.						

进一步研究^[30]^发现，在放射敏感的*EGFR*突变型NSCLC细胞HCC827中，吉非替尼可通过减少ERK1/2和AKT的磷酸化，阻断其介导的抗凋亡及促增殖作用，从而进一步增强放射敏感性。CALEB 30106研究结果提示在局部晚期不可切除的NSCLC患者中，序贯化放疗中使用吉非替尼疗效较好（中位PFS为13.4个月，中位OS达19个月），而分层分析却未显示*EGFR*的突变状态与生存预后的相关性^[31]^，但是该试验样本量太少（13例突变阳性），此结论需进一步研究探讨。目前尚无更多比较TKI联合放疗在不同*EGFR*突变状态NSCLC中疗效的研究报道。

## 4 问题与展望

虽然目前研究提示*EGFR*突变人群对放疗敏感，但未显示出*EGFR*突变与放疗疗效、生存预后的明显相关性。由于大部分的NSCLC患者在TKI治疗后产生继发耐药，挽救性放疗可能为这部分患者带来一定的生存获益，但其放射敏感性在TKI继发耐药后是否发生变化尚不清楚。

尽管众多研究肯定了TKI联合放疗在部分NSCLC患者的安全及有效性，但多为小样本的回顾性研究，偏倚因素较多，其疗效尚需大型Ⅲ期随机对照试验进行验证。此外，针对*EGFR*突变人群，放疗联合TKI治疗的疗效值得进一步探索。但是，联合治疗可能加重放疗及靶向治疗的副反应，如皮疹、腹泻、放射性肺炎、骨髓抑制、食管炎等，因此，TKI联合放疗在NSCLC治疗中仍需谨慎对待。寻找有效预测放疗疗效分子靶标，为患者实施个体化的放疗计划，使NSCLC患者的生存期得到突破是我们努力的方向。
